# How the Sea Salt Chocolate Cookie Became a Thing: The Role of Flavor Pairing in Obesity

**DOI:** 10.1002/oby.70285

**Published:** 2026-09-01

**Authors:** Mohammed Ayyad, Eugene Kim

**Affiliations:** ^1^ Department of Medicine Rutgers New Jersey Medical School Newark New Jersey USA

## Abstract

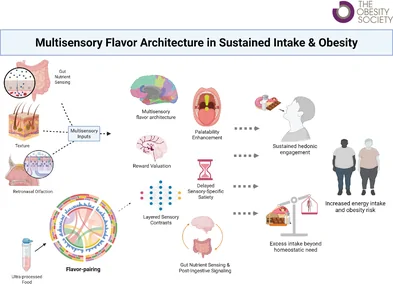

## Graphical abstract

Proposed framework illustrating how flavor pairing and multisensory flavor architecture may sustain hedonic eating through enhanced palatability, reward preservation, delayed sensory‐specific satiety, and post‐ingestive signaling, thereby promoting excess energy intake and obesity risk.

## 1 Introduction

The widespread emergence of flavor‐paired foods reflects more than culinary creativity; it represents an implicit understanding of how contrasting sensory signals sustain eating behavior. While hyperpalatable foods are typically framed in terms of macronutrient composition or energy density, the multisensory architecture underlying flavor pairing remains underexplored as a driver of overeating. This Perspective proposes that flavor pairing may represent one component of a broader sensory architecture that sustains intake by preserving palatability, modulating aversive taste signals, delaying sensory‐specific satiety, and increasing sensory variety within or across eating episodes. Through contrast‐driven reward preservation amidst the broader sensory architecture, we argue that it might promote hedonic engagement, thereby prolonging eating independent of metabolic hunger.

Here, we refer to taste as gustatory qualities such as sweet, salty, bitter, sour, and umami. Flavor, by contrast, is a broader multisensory percept constructed from taste, retronasal olfaction, texture, chemesthesis, temperature, visual appearance, and contextual cues during the experience of eating and formed from the processing of these signals in brain regions. The convergence and integration of sensory inputs within these regions shape the valuation and decision‐making processes that govern eating behavior. Dopaminergic reward signaling activated by layered gustatory stimuli, particularly when incorporated within palatable flavor matrices, can reinforce food consumption even when homeostatic energy requirements have been met, an effect that is especially pronounced in environments rich in engineered hyperpalatable foods. The sea salt chocolate cookie is useful as an example. Salt in combination with chocolate does not function solely by adding a consciously salty taste. In bitter food matrices such as cacao or coffee, sodium suppresses bitterness, thereby increasing the perceptual salience of sweetness while moderating peak sweet intensity [1]. Thus, the appeal of salted chocolate likely emerges from the interplay of flavor pairing, sweet‐salty contrast, and subtler taste‐taste interactions. This is supported by Breslin and Beauchamp demonstrating how salt can selectively suppress bitterness more than sweetness, thereby increasing the relative salience of sweet taste within bitter–sweet mixtures [1]. This mechanism may help explain why sodium is frequently incorporated into bitter food matrices such as cacao, coffee, and certain vegetables despite minimal conscious perception of saltiness. Similarly, other taste–taste interactions occur across gustatory combinations including sweet–bitter, sweet–sour, salty–umami, and other pairings in which one taste quality modulates the perception of another, suggesting that gustatory interactions may enhance palatability, increase flavor complexity, and sustain reward‐driven consumption beyond the effects of any individual taste quality in isolation. The mechanisms through which flavor pairing may influence food intake likely extend beyond any single gustatory interaction and involve multiple sensory, neural, behavioral, and post‐ingestive pathways. A proposed framework summarizing these interacting mechanisms is shown in Table 1.

**TABLE 1:** Mechanistic framework linking flavor pairing to sustained intake and obesity.

Mechanistic domain	Core concept	Proposed implication for overeating
Multisensory Flavor Integration	Flavor arises from integration of taste, retronasal olfaction, texture, chemesthesis, temperature, and contextual cues within high‐order reward regions.	Layered sensory inputs may maintain hedonic engagement beyond metabolic need.
Taste–Taste and Flavor Interactions	Interactions among sweet, salty, bitter, sour, and umami qualities can alter perceived intensity, palatability, and flavor balance.	Flavor pairing may increase palatability by reducing aversive sensory signals and enhancing desirable sensory qualities.
Sensory‐Specific Satiety	Repeated exposure to the same flavor decreases pleasantness and neural reward responses.	Flavor contrast may delay satiety‐driven disengagement from eating.
Variety‐Driven Intake and Hedonic Contrast	Introducing new sensory or flavor stimuli renews reward value and may increase intake.	Alternating or paired flavors may sustain consumption by repeatedly renewing hedonic valuation.
Olfactory and Non‐Gustatory Contributions	Sensory‐specific satiety operates across odor and other sensory domains independently of taste.	Flavor pairing may maintain intake through many sensory inputs such as olfactory novelty even when gustatory stimulation remains constant.
Ultra‐Processed Food Sensory Architecture	Many ultra‐processed foods incorporate multiple engineered sensory contrasts simultaneously.	Layered flavor, texture, and aroma contrasts may contribute to sustaining reward signaling and delay satiation.
Post‐Ingestive Reinforcement (Appetition)	Nutrients can condition flavor preferences and stimulate intake through post‐oral gut‐brain signaling independent of oral sensory stimulation.	Paired flavors may become reinforced through both sensory reward and post‐ingestive nutrient feedback, possibly sustaining hedonic engagement.
Variety Restriction Interventions	Reducing sensory variety may decrease intake of energy‐dense foods.	Reduced sensory contrast may accelerate sensory‐specific satiety.
GLP‐1 and Reward Modulation	GLP‐1 receptor activation attenuates neural responses to food reward and craving.	GLP‐1 receptor activation may restore meal termination by reducing contrast‐driven hedonic reinforcement and modulating appetition among many other mechanisms.

## 2 The Role of Flavor Pairing in Modulating Sensory‐Specific Satiety

Another central mechanism governing meal termination is sensory‐specific satiety. As a particular flavor is consumed repeatedly, its pleasantness declines, facilitating cessation of intake. Neuroimaging studies demonstrate that sensory‐specific satiety is associated with reduced orbitofrontal cortex activation to a recently consumed food or flavor while neural responsiveness to alternative flavors remains preserved [2, 3]. Consistent with this, Kringelbach et al. demonstrated declining orbitofrontal cortex activity correlating with the subjective loss of pleasantness during consumption of a certain food, providing a neural correlate of sensory‐specific satiety [4]. Thus, introducing sensory contrast through flavor pairing may disrupt this attenuation, renewing reward value and prolonging engagement by generating “micro‐variety” and continuous sensory renewal within a single eating episode. Notably, the sensory unit in sensory‐specific satiety is not affected by taste alone. Le Magnen demonstrated that rats consumed more when the same diet was presented in different odorized forms, while Rolls and colleagues showed that olfactory sensory‐specific satiety can occur independently of gustatory stimulation [5, 6]. Together, these findings suggest that sensory‐specific satiety may operate at the level of the broader multisensory flavor experience including retronasal olfaction and chemesthetic cues rather than isolated taste, allowing renewed intake despite nutritional sufficiency. Based on these remarks, we suspect that temporal alternation between differing sensory profiles may further amplify this effect by repeatedly reintroducing novelty into the eating experience. In this context, alternating between different flavor stimuli may sustain intake by shifting sensory contrasts, thereby counteracting sensory‐specific satiety and renewing hedonic intake.

Quantitative experimental data support the role of sensory contrast in increasing food intake. In a randomized within‐subject laboratory study conducted over three weekly sessions, participants consumed a standardized meal under monotonous conditions (no condiments), simultaneous variety (condiments available throughout), or successive variety (condiments introduced sequentially after intake of the plain food had ceased). Energy intake increased by 24% with simultaneous variety and 41% with successive variety compared to monotonous, consistent with variety‐driven delay of sensory‐specific satiety [7]. Similarly, Hetherington and colleagues reported higher total energy intake when a food with a different sensory or taste profile was introduced during snacking, with incongruent tastes producing increases of up to 40% [8]. Furthermore, Yeomans et al. demonstrated that alternating salted and unsalted foods increases intake, particularly when a palatable item follows a bland one, suggesting that hedonic contrast enhances liking and promotes additional consumption [9]. These findings suggest that contrast‐driven renewal of reward, whether through gustatory contrast or broader flavor variation, may sustain eating beyond physiological need, representing a plausible sensory mechanism promoting overeating.

Clinically, sensory architecture may represent a modifiable target for earlier meal termination. While most dietary interventions focus on caloric restriction or macronutrient composition, limiting sensory variety within energy‐dense foods promotes monotony, reduces intake within the targeted domain, and modestly decreases overall excess energy consumption, likely by accelerating sensory‐specific satiety [10]. Supporting this concept, short‐term trials demonstrate that increasing sensory variety within a meal increases energy intake by approximately 22% compared with single‐food conditions, with increases of roughly 50–60 kcal/day per additional food offered [11]. In a real‐world randomized trial, Hall and colleagues demonstrated approximately a 500 kcal/day increase in ad libitum energy intake with an ultra‐processed diet compared with a nutritionally matched unprocessed diet, resulting in measurable weight gain within 2 weeks [12]. Although specific flavor pairings were not independently assessed, many ultra‐processed foods incorporate structured sensory contrasts that may sustain reward signaling and delay satiety. Such contrasts are often deliberately engineered across multiple sensory domains simultaneously, including sweetness, saltiness, aroma intensity, texture heterogeneity, temperature contrast, and rapid flavor transitions during mastication. These layered sensory properties may collectively maintain hedonic engagement throughout eating episodes. Whether overeating is correlated to specific gustatory contrasts by flavor pairing or broader hyperpalatable food design that incorporates aroma, texture, chemesthesis, and layered sensory stimulation remains unclear. Nevertheless, flavor pairing may represent one component of contrast‐driven sensory architecture warranting further evaluation.

Additionally, flavor pairing's influence on intake is unlikely to be solely driven by sensory‐specific satiety. Food‐reward studies provide evidence that consumption is shaped not only by the oral sensory properties but also by the post‐oral nutrient consequences. Sclafani and Ackroff summarized the significant evidence that gut nutrient sensing can stimulate appetite and drive flavor preferences. This post‐oral reinforcement is often described as “appetition,” in which nutrients such as glucose condition flavor preferences and promote intake independent of oral sweetness alone [13, 14]. Accordingly, sweet‐containing foods may reinforce eating through both sensory reward and post‐ingestive nutrient signaling. Experimental evidence suggests post‐oral sugar consumption can promote positive feedback signals via post‐ingestive signaling, likely through SGLT1/SGLT3 receptors that increase reward value beyond sweet taste alone, thereby driving contrasting flavor preferences and stimulating appetite [15].

## 3 Glucagon‐Like Peptide‐1 (GLP‐1) Signaling as a Potential Modulator of Sensory‐Specific Satiety

Because sensory contrast may prolong intake by sustaining hedonic reward signaling and delaying sensory‐specific satiety, pathways that modulate reward valuation may help restore meal termination. Incretin signaling, particularly via GLP‐1, links gut–brain satiety regulation with neural circuits governing hedonic eating. Beyond hypothalamic and brainstem pathways, GLP‐1 receptor activation influences mesolimbic reward regions which regulate reward valuation and craving [16]. Neuroimaging studies demonstrate that GLP‐1 receptor agonists attenuate activation in reward and salience networks during exposure to highly palatable food cues, and randomized controlled trials (RCTs) with liraglutide and exenatide demonstrate reduced neural responses to anticipated food reward alongside decreases in hunger and cravings [16, 17, 18]. A systematic review of RCTs evaluating the effects of GLP‐1 receptor agonists reported significant reductions in hunger and food preoccupation, decreased cravings for sweet, salty, fatty, and savory foods, and reduced liking and wanting for high‐fat foods. This suggests that GLP‐1 signaling may influence both homeostatic and hedonic drivers of eating behavior through effects on both central reward processing and post‐ingestive signaling [19]. Because reward valuation dynamically changes throughout a meal, modulation of mesolimbic and orbitofrontal processing by GLP‐1 receptor agonists may accelerate the decline in hedonic responsiveness that contributes to sensory‐specific satiety and appetition, though these mechanisms remain theoretical.

## 4 Conclusion

Flavor pairing illustrates how contrast within the broader sensory architecture of food may sustain hedonic engagement, modulate aversive sensory signals, and delay sensory‐specific satiety. Prolonged intake may reflect contrast‐driven sensory reward dynamics acting alongside post‐ingestive reinforcement rather than hunger alone, highlighting sensory architecture as an underrecognized contributor to obesogenic environments. Future research should evaluate flavor pairing as an independent variable by comparing individual flavor stimuli with paired flavor combinations. For example, studies could compare flavor A, flavor B, and flavor A + B conditions to assess their effects on palatability, sensory‐specific satiety, reward signaling, food intake, and long‐term dietary outcomes. Studies should also consider evaluating whether and how emerging gut–brain therapeutics, such as GLP‐1 receptor agonists, modulate sensory‐specific satiety, reward valuation, and appetition to limit excessive intake and identify additional targets that enhance sensory‐specific satiety and accelerate meal termination signals in obesity management. For clinicians, this framework may encourage greater attention to sensory composition and flavor pairing when counseling patients about eating patterns and highlights modifiable features of food design as potential intervention targets to improve hedonic control of food intake and support sustainable weight management.

## Author Contributions

The authors were solely responsible for all aspects of this work, including conceptualization, literature review, analysis, drafting, and final approval of the manuscript.

## Funding

The authors have nothing to report.

## Conflicts of Interest

The authors declare no conflicts of interest.

## Data Availability

The data that support the findings of this study are available from the corresponding author upon reasonable request.
